# Self-medication profile of ENT patients

**DOI:** 10.1016/S1808-8694(15)30038-0

**Published:** 2015-10-19

**Authors:** Alexandre Barbosa Servidoni, Liliane Coelho, Marcos de Lima Navarro, Fernanda Gobbi de Ávila, Raquel Mezzalira

**Affiliations:** a 3^rd^ year resident in otolaryngology; b Otolaryngologist; c Otolaryngologist – MSc. ENT Department - Penido Burnier Institute

**Keywords:** self-medication, otorhinolaryngology, otorhinolaryngological patients

## Abstract

**Aim:**

To describe self-medication habits of patients with otorhinolaryngological disorders to characterize problematic aspects in order to manage and correct them.

**Study Design:**

Descriptive.

**Materials and Methods:**

A multiple-choice questionnaire was used to obtain the data, consisting of 17 questions about self-medication habits. It was distributed to the patients assisted in our ENT clinic, in July/2003.

**Results:**

Approximately 83% of the admitted patients have practiced self-medication (without medical prescription). However, 73% of them stated that it wasn’t mandatory to present a prescription in order to obtain the medicine. The most utilized drugs were: analgesics/antipyretics (90%), cold and flu drugs (78%), and NSAIDs (69%); antibiotics were the 8th (11%). Among the reasons or diseases patients believed to suffer from that justified the self-medication practice, we observed: headaches (76%), cold/flu (74%) and nonspecific febrile illnesses (56%); otitis, in general, was the last (12%).

**Conclusions:**

This study demonstrates the need for continuous educational programs about the risks of self-medication, besides appropriate governmental regulation and inspection.

## INTRODUCTION

Self-medication is a common habit in our country, mostly for patients with otolaryngological disorders, and it has always been a very controversial and debated issue in doctor-patient and pharmaceutical relationships. Especially in developing countries, such as Brazil, self-medication may be considered a need that complements our health care system. Thus, the World Health Organization published guidelines to assess the medications that could be bought over the counter, without a physician's prescription^1^. A wrongly taken medication may bring dire consequences to the individual, such as masking developing diseases, iatrogenic problems and many other undesirable effects. In our country, where most of the population is poorly educated, especially about medications and their correct use, self-medication is even riskier. We still lack rigorous controls set up by regulating agencies, and we have a very weak involvement of health care professionals in educating their patients. Besides, the law about the need to present a medical prescription to buy a medication is not strongly enforced in our country, thus in Brazil we have about 80 million people who regularly by medication over the counter and self medicate, according to data from the Brazilian Association of Pharmaceutical Companies^2^. In this paper we present a descriptive study on self medication practice by patients seen at the Otolaryngology Department of the Penido Burnier Institute. The goal of this paper is to describe self medication habits of patients seen in our clinic, in order to characterize and quantify disorders that may be corrected.

## MATERIALS AND METHODS

We used a questionnaire with multiple choice questions to obtain our data (Attachment 1), with 17 questions about self medication habits, which was answered by the patient during his/her wait for the medical consult, after being duly informed about its aim. This questionnaire was prepared based on a paper from Arrais et al. (1997)^3^ and with suggestions made by the professionals of our institution. We distributed 150 questionnaires equally among regular patients during week days and on weekend emergency visits. The study was carried out during the month of July, 2003.

## RESULTS

**Graph 1:**
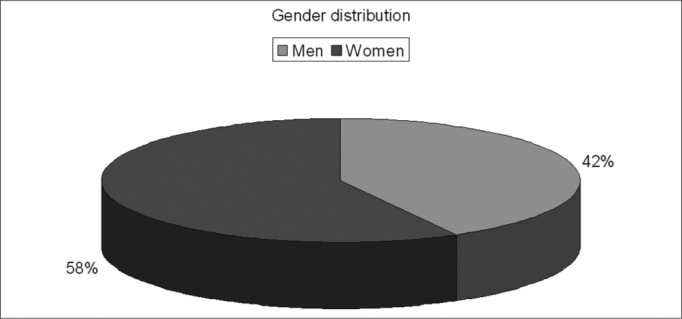
Gender distribution.

**Graph 2:**
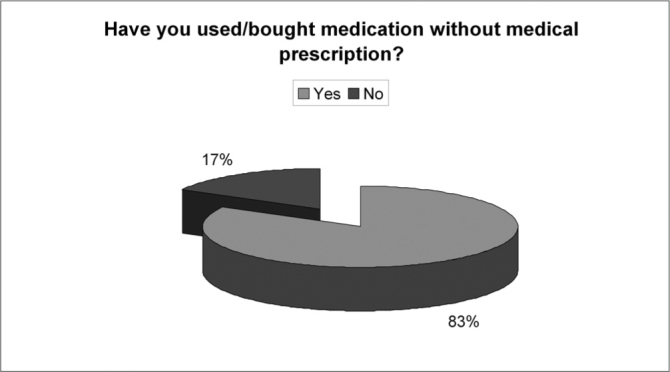
No medical prescription.

**Graph 3:**
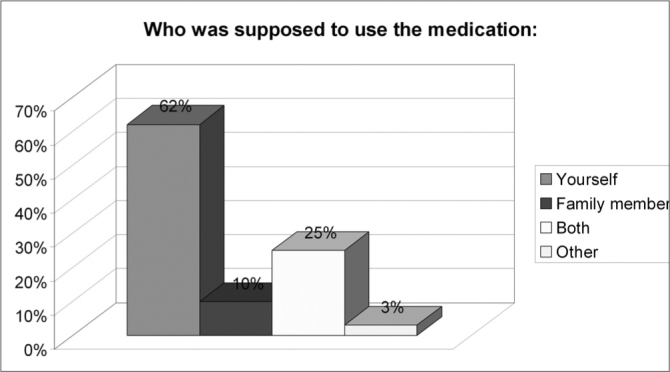
Medication use.

**Graph 4:**
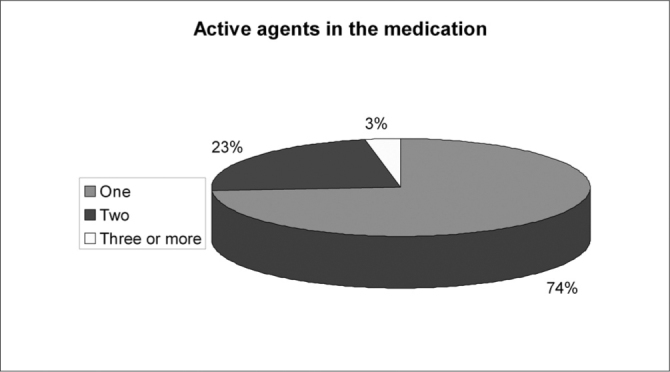
Active agents.

**Graph 5:**
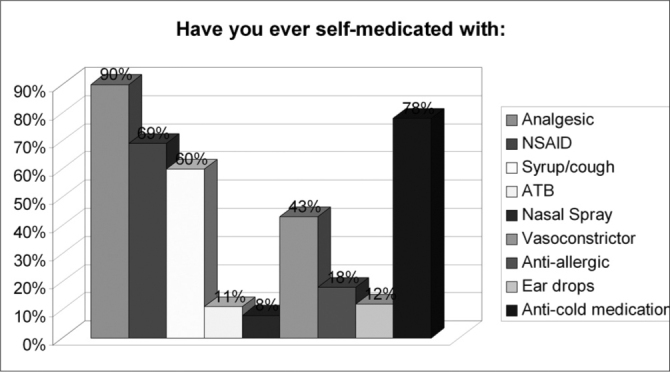
Medication used.

**Graph 6:**
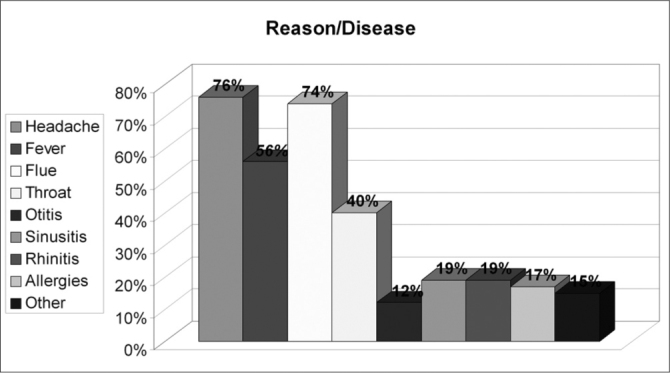
Reasons/ diseases.

**Graph 7:**
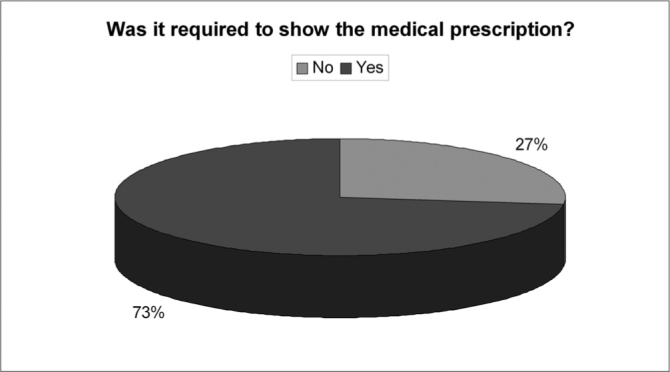
Medical prescription required.

Of the 150 questionnaires distributed, 72 (48%) were answered, and the rest was probably ignored by the patients. Of the 17 questions presented, we noticed a certain difficulty in answering only the one related to the amount of active agents present in the medication used, despite all the explanation provided. Over half of the patients (57%) did not answer that question. Age ranged between 15 and 72 years, with an average of 38 years. Gender distribution was of 58% women and 42% men. Almost 83% of the users had used or bought medication without a medical prescription. Notwithstanding, of this total, 73% answered the medication they used did not require a medical prescription to be bought. 62% of the time the medication was for their own use; 10% for family members; 25% for both situations and 3% bought it for other people. 72% took advice from the drug store pharmacist or sales person and 54% from third parties (relative, neighbor or friend). Besides, 56% of the patients had suggestions from the drugstore attendant when buying the medication. Among those who could answer the question, 11.74% reported that there was only 1 active agent in the medication; 23% reported 2 active agents and 3% reported 3 or more. As to most used medications we obtained the following results: first and foremost analgesic and antipyretic (90%), followed by cold symptoms medication (78%) and anti-inflammatory (69%). Antibiotic agents came next to the last (11%). (Table 1). As for the reasons or motives that patients believed they had to justify self-medication, our study showed headaches in first place (76%), followed by cold and flue symptoms (74%) and fever (56%). Otitis, for instance, came last (12%). (Table 2) About 51% of the interviewees based themselves in old prescriptions they had at home. The remaining questions in the questionnaire were not relevant for the final goal of this study. Graph 1, Graph 2, Graph 3, Graph 4, Graph 5, Graph 6, Graph 7 show the results of the present study.

**Table 1:** Medication - Incidence (%)

Analgesic/antipyretics	90%
Anti-inflammatory	69%
Cough syrups	60%
antibiotics	11%
Nasal steroids (nasal sprays)	8%
Decongestants/vasoconstrictors	43%
Anti-allergic/antihistaminic drugs	18%
Ear drops	12%
Anti-cold medication	78%

**Table 2:** Symptoms - Incidence (%)

Headaches	76%
Fever	56%
Cold/flue	74%
Throat inflammations/infections	40%
Otitis	12%
Sinusitis	19%
Rhinitis	19%
Allergies	17%
Other	15%

## DISCUSSION

Our study showed that women self-medicated more than men, especially those in the third and fourth decades of life; and men had a more homogenous distribution in the different ages analyzed, according to results attained by Carlini5 and Paulo8. We noticed that most of the medication was for self use, and not for family members; such fact reduces the risk of crossed contamination of relatives by shared use of medication (such as nasal drops), as well as it reduces the risks of inadequate and/or incomplete for other family members. Such finding had already been reported by Arrais et al3. As for the problems that led the patients to self-medicate, most of them were self-limited diseases, and this does not justify the use of antibiotics. Notwithstanding, the use of analgesic, cold control medications and cough syrups, if properly used, would be justified when aiming at improving the patient's life quality and symptom relieving. Many of such medication still reflect the national market, characterized by the presence of many unnecessary products, or at least, products of questionable efficacy, intensely advertised, but without due public education. We also noticed a reasonably low number of medications that did require a medical prescription to be sold, and such fact may reflect a greater populational awareness in regards of regulations and the very dangers of wrong self-medication. Finally, we noticed that medications are incorporated in the very dynamics of a consumption society, and thus, subject to market interest and setting them away from their prime objective of prevention and treatment of human diseases.

Attachment 1 **Questionnaire model**

Penido Burnier Institute

This questionnaire is part of a study about self-medication. This study is being developed at the otolaryngology department of this institution and aims at listing the main medications used by the population without a medical prescription. The results of this paper, based on your answers will be presented in medical meetings and published in medical journals. Thus, if you agree with the terms of this survey, please answer the questionnaire below (you do not have to write your name) and hand it back to the receptionist. We thank you for your help.

Identification

Gender: male ( ) female ( )

Age:

Marital status: single ( ) married ( ) widow (er) ( ) Divorced/Separated ( ) Other ( )

Questionnaire

1) Have you ever used or bought medication without a medical prescription?

( ) YES( ) NO

2) Who was going to use the medication?

( ) yourself

( ) another family member

( ) both

( ) someone else

3) Did you forget or lost the prescription at the time of purchase?

( ) YES( ) NO

4) Have you ever taken advice from the pharmacist or sales person in order to purchase medication?

( ) YES( ) NO

5) Have you ever received unsolicited advice (in the drugstore) ?

( ) YES( ) NO

6) Have you taken advice from third parties?

( ) YES( ) NO

7) If affirmative (previous question), from whom?

( ) neighbor ( ) relative

( ) friend ( ) someone else

8) Have you ever used old medical prescriptions?

( ) YES ( ) NO

9) If affirmative, these past prescriptions were:

( ) yours ( ) from someone else - who:

10) The medication bought/used required a medical prescription by law?

( ) YES ( ) NO

11) How many active agents (salt/substance/generic) were there in the drug?

( ) 01 active agent ( ) 02 active agents ( ) 03 or more

12) Mark which medications you have already self-medicated with:

( ) analgesics/antipyretics

( ) anti-inflammatory agents

( ) cough syrups

( ) anti-asthma drugs

( ) antibiotics

( ) systemic steroids (oral)

( ) nasal steroids (nasal sprays with steroids)

( ) nasal decongestants/vasoconstrictors

( ) anti-allergic/anti-histaminic

( ) ear drops

( ) cold/flue control medications

( ) other - which:

13) Which reasons/diseases below you believed you had?

( ) headache

( ) fever

( ) cold/flue

( ) throat inflammations / infections (pharyngitis, tonsillitis, laryngitis)

( ) ear infections/ inflammations (otitis)

( ) sinusitis

( ) rhinitis

( ) allergies

( ) oral lesions

( ) skin lesions

( ) other head and/or neck disorders

( ) reflux

( ) pulmonary diseases

( ) other - which:

14) For how long did you use the medication?

( ) 01 day

( ) 02 days

( ) 03 to 05 days

( ) over 5 days – how many?

15) Followed the label instructions?

( ) YES( ) NO

16) When was your last medical visit?

( ) less than 01 week ago

( ) between 01 week and 01 month ago

( ) between 01 and 03 months ago

( ) over 3 months ago - When:

( ) I don’t remember

17) This space is for comments / criticism / suggestions that you may have:

## CONCLUSION

This paper shows the need to carry out educational campaigns to alert the population about the use of many medications available in the market. For that, it is paramount to have an active participation of health care professionals, specially physicians and pharmacists, besides the help from the pharmaceutical industry, government regulations and broad and continuous inspection by the competent authorities.
